# Functional Consequences of PDK4 Deficiency in Doberman Pinscher Fibroblasts

**DOI:** 10.1038/s41598-020-60879-6

**Published:** 2020-03-03

**Authors:** Luiz Bolfer, Amara H. Estrada, Chelsea Larkin, Thomas J. Conlon, Francisco Lourenco, Kathryn Taggart, Silveli Suzuki-Hatano, Christina A. Pacak

**Affiliations:** 1 Department of Small Animal Clinical Sciences, University of Florida College of Veterinary Medicine, Gainesville, FL 32610 USA; 2 CR Scientific and Compliance Consulting, LLC, Gainesville, FL 32608 USA; 3 Department of Pediatrics, University of Florida College of Medicine, Gainesville, FL 32610 USA; 4 Department of Molecular Genetics and Microbiology, University of Florida College of Medicine, Gainesville, FL 32610 USA

**Keywords:** Mitochondria, Molecular biology

## Abstract

A splice site mutation in the canine pyruvate dehydrogenase kinase 4 (*PDK4*) gene has been shown to be associated with the development of dilated cardiomyopathy (DCM) in Doberman Pinchers (DPs). Subsequent studies have successfully demonstrated the use of dermal fibroblasts isolated from DPs as models for PDK4 deficiency and have shown activation of the intrinsic (mitochondrial mediated) apoptosis pathway in these cells under starvation conditions. For this study, we sought to further explore the functional consequences of PDK4 deficiency in DP fibroblasts representing PDK4^wt/wt^, PDK4^wt/del^, and PDK4^del/del^ genotypes. Our results show that starvation conditions cause increased perinuclear localization of mitochondria and decreased cell proliferation, altered expression levels of pyruvate dehydrogenase phosphatase (PDP) and pyruvate dehydrogenase (PDH), dramatically increased PDH activity, and an impaired response to mitochondrial stress in affected cells. In sum, these results show the broad impact of PDK4 deficiency and reveal mechanistic pathways used by these cells in an attempt to compensate for the condition. Our data help to elucidate the mechanisms at play in this extremely prevalent DP disorder and provide further support demonstrating the general importance of metabolic flexibility in cell health.

## Introduction

Dilated Cardiomyopathy (DCM) is one of the most prevalent causes of heart disease in humans and canines. More than 30 genetic mutations and transcriptional alterations related to various molecular and biochemical pathways have been linked to human and canine DCM^1–17^. Specifically, the Doberman Pinscher (DP) canine breed is highly susceptible to the development of non-ischemic DCM with high mortality rates.

Recent studies have shown that for many DPs, DCM can be attributed to either 1) a splice site deletion in the pyruvate dehydrogenase kinase 4 (*PDK4*) gene, 2) a missense variant in the titin (*TTN*) gene, or 3) an unfortunate combination of both mutations in the same dog^6,8^. The newly described titin variant is predicted to change the protein’s structure and has been shown to decrease active tension in myofibers from affected DPs. As titin is the gene most commonly associated with DCM in humans, its functional importance as a molecular spring in muscle and its roles in biochemical sensing and signaling have been well described^18–23^.

In this study, we aimed to evaluate the biochemical and molecular mechanisms that are involved in PDK4 deficiency in DPs. Primary dermal fibroblasts from healthy DPs (control - PDK4^wt/wt^) and DPs carrying the PDK4 mutation (both heterozygous - PDK4^wt/del^, and homozygous - PDK4^del/del^) have been previously examined and shown to have an increased susceptibility to apoptosis activation under starvation conditions that was mitigated with adeno-associated virus (AAV) mediated gene delivery of healthy *PDK4* to affected cells^24,25^. In the present study, we sought to perform comparative analyses between fibroblasts representing the three genotypes to gain a more complete understanding of the mechanistic pathways involved in the cellular response to this deficiency. Previous studies have shown that PDK4 can be upregulated by lipids, for example, those derived from decanoic acid and that PDK4 expression can be regulated by both the PPARβ/δ and TGFβ signaling pathways^26–28^. These studies demonstrate that fibroblasts are excellent models for the assessment of PDK4 function. To evaluate PDK4 deficiency in DP fibroblasts, cells were evaluated for cell morphology and mitochondrial localization within cells, PDK expression levels, pyruvate dehydrogenase (PDH) activity and abundance, and response to mitochondrial stress based upon oxygen consumption analyses. Through phosphorylation of PDH, PDK4 serves as an important regulator of mitochondrial fuel usage by switching away from glucose oxidation under conditions of low glucose availability. To better understand this disorder at the molecular level, the present study evaluated healthy and deficient cells in their response to glucose starvation. In doing so, we have helped to identify the broader mechanistic consequences and compensatory pathways at play in PDK4 deficiency.

## Results

### Starved primary dermal fibroblasts require PDK4 to survive

**Figure 1:**
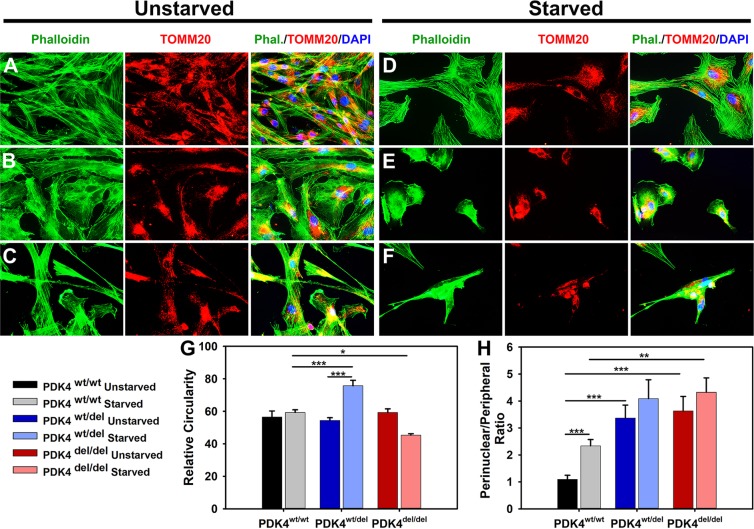
DP fibroblast cellular morphology and mitochondrial localization. (**A–C**) Primary dermal fibroblasts from healthy controls PDK4^wt/wt^, heterozygous PDK4^wt/del^, or homozygous PDK4^del/del^, DPs were evaluated with IF staining of phalloidin (green) to reveal overall cellular architecture and the mitochondrial outer membrane protein TOMM20 (red) to locate mitochondria within cells. (**D–F**) Cells representing the three different genotypes were exposed to 24 hours of starvation conditions. (**G**) A graph indicating how relative circularity changed in both PDK4^wt/del^ (increased) and PDK4^del/del^ (decreased) cells in response to starvation conditions as compared to healthy controls under the same conditions. (**H**) Perinuclear localization of mitochondria was increased in both PDK4^wt/del^ and PDK4^del/del^ cells as compared to controls under the same condition except there was no significant difference between starved PDK4^wt/del^ and PDK4^wt/wt^ cells. (Data presented as mean + std. err. *p < 0.05, **p < 0.01, ***p < 0.001).

In order to evaluate the effects of glucose deprivation in the context of PDK4 deficiency, we placed robust primary dermal fibroblast cultures representing PDK4^wt/wt^, PDK4^wt/del^, and PDK4^del/del^ genotypes into culture medium that lacked glucose. After 24 hours of starvation, cells were evaluated for differences in general cell morphology and mitochondrial localization as compared to unstarved cells representing the same genotypes **(**Fig. 1**)**. Immunofluorescence staining of f-actin (phalloidin) in fixed cells revealed that PDK4^wt/wt^ fibroblasts exhibit no significant changes in morphology after 24 hours of starvation **(**Fig. 1A,D,G**)**. In contrast, fibroblasts representing the PDK4^wt/del^ **(**Fig. 1B,E**)** and PDK4^del/del^ **(**Fig. 1C,F**)** genotypes displayed significant changes in cellular circularity as compared to controls under starvation conditions with a significant increase observed in PDK4^wt/del^ cells and a significant decrease observed in PDK4^del/del^ cells **(**Fig. 1D–F,G**)**.

These evaluations also showed that general cellular abundance was significantly reduced in response to starvation in both PDK4^wt/del^ and PDK4^del/del^ cells as compared to PDK4^wt/wt^ controls (PDK4^wt/del^ 36% ± 2 and PDK4^del/del^ 25% ± 1; p < 0.05 [% of PDK4^wt/wt^]) while there was no significant difference between cells in unstarved conditions. Assessment of ratios of perinuclear to peripheral mitochondrial localization showed a significant increase in both PDK4^wt/del^ and PDK4^del/del^ cells as compared to healthy controls in unstarved conditions **(**Fig. 1H**)**. Under starvation conditions, only PDK4^del/del^ cells showed significantly more perinuclear localization of mitochondria as compared to controls but PDK4^wt/del^ cells also showed an increasing trend. These observations support previous findings that PDK4 function is required for healthy cell morphology and viability in response to starvation.

### PDK4 deficiency alters PDK transcription profiles

**Figure 2:**
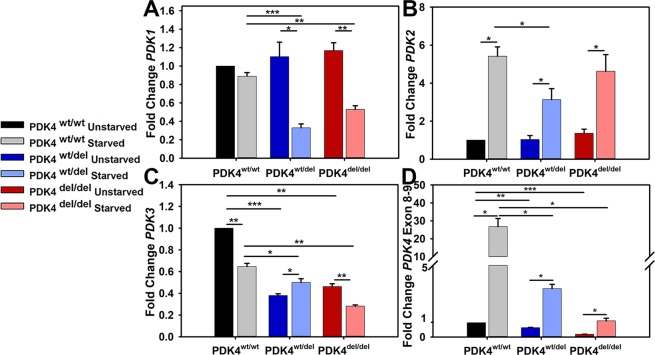
PDK isoform transcription levels in response to starvation. Relative transcription levels of (**A**) *PDK1*, (**B**) *PDK2*, (**C**) *PDK3*, and (**D**) *PDK4* in cells representing each of the three groups PDK4^wt/wt^, PDK4^wt/del^, and PDK4^del/del^ cells in unstarved and starved conditions. (Data presented as mean + std. err. *p < 0.05, **p < 0.01, ***p < 0.001).

As there are a total of 4 different PDK isoforms, we sought to determine how cells representing the three different genotypes differed in their PDK transcription levels under typical culture conditions and how this profile may be altered under glucose-free (starvation) conditions. *PDK1* transcript levels were similar across all three genotypes under typical culture conditions and remained unchanged in response to 24 hours of starvation in PDK4^wt/wt^ cells. In contrast, *PDK1* transcript levels were significantly reduced following starvation in both PDK4^wt/del^ and PDK4^del/del^ cells as compared to unstarved controls **(**Fig. 2A**)**. *PDK2* transcript levels were similar across all three genotypes under typical culture conditions and were significantly increased in response to 24 hours of starvation in all fibroblasts as compared to unstarved conditions **(**Fig. 2B**)**. *PDK3* transcript levels were significantly reduced in both PDK4^wt/del^ and PDK4^del/del^ cells under typical culture conditions as compared to PDK4^wt/wt^ cells. In response to 24 hours of starvation, *PDK3* remained significantly reduced in both PDK4^wt/del^ and PDK4^del/del^ cells as compared to PDK4^wt/wt^ cells. When compared to corresponding unstarved culture conditions, *PDK3* transcript levels were significantly reduced in fibroblasts representing PDK4^wt/wt^ and PDK4^del/del^ genotypes. In contrast, *PDK3* transcript levels were significantly increased in the PDK4^wt/del^ cells as compared to unstarved controls **(**Fig. 2C**)**.

Finally, *PDK4* transcript levels were examined using a primer-probe set that would recognize the exon 8–9 boundary of PDK4 that should be present in transcripts derived from all three genotypes as this area is located upstream of the splice site deletion present in the affected cells between *PDK4* exons 10 and 11. *PDK4* was significantly decreased in the PDK4^wt/del^ and PDK4^del/del^ cells under normal culture conditions as compared to PDK4^wt/wt^ cells. In contrast, *PDK4* transcription was significantly increased in response to 24 hours of starvation in fibroblasts representing all three genotypes as compared to unstarved conditions but this effect was far more profound in the PDK4^wt/wt^ cells than either of the PDK4 deficient lines **(**Fig. 2D**)**.

### PDK4 deficiency alters PDP and PDH transcription levels

**Figure 3:**
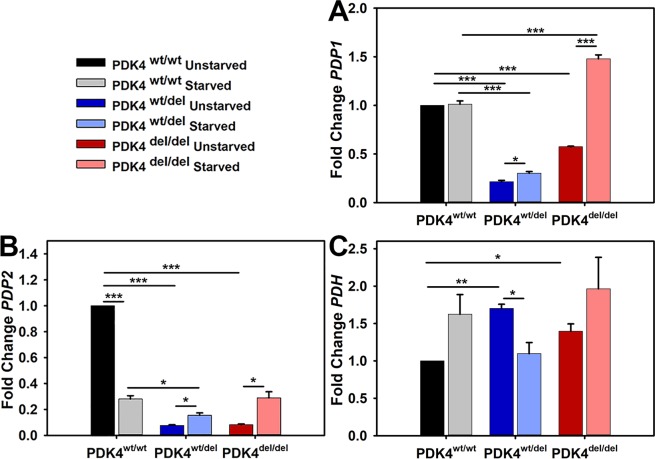
PDP isoforms 1 and 2 and PDH transcription levels in response to starvation. Relative transcription levels of (**A**) PDP1, (**B**) PDP2, and (**C**) PDH, in cells representing each of the three groups PDK4^wt/wt^, PDK4^wt/del^, and PDK4^del/del^ cells in unstarved and starved conditions. (Data presented as mean + std. err. *p < 0.05, **p < 0.01, ***p < 0.001).

To determine the impact of PDK4 deficiency on pyruvate dehydrogenase phosphatase (PDP) and pyruvate dehydrogenase (PDH) transcription under normal and 24 hours of starvation culture conditions, gene expression assays were performed to evaluate relative levels of *PDP1* and *PDP2* in cells representing all three genotypes. *PDP1* expression levels were significantly higher in PDK4^wt/wt^ cells under normal culture conditions as compared to the deficient lines. *PDP1* levels were unchanged in PDK4^wt/wt^ cells under starvation conditions as compared to unstarved controls but significantly increased in both PDK4^wt/del^ and PDK4^del/del^ cells as compared to their respective *PDP1* levels in normal culture conditions **(**Fig. 3A**)**. In contrast, *PDP2* expression levels were significantly increased in PDK4^wt/wt^ cells under normal culture conditions as compared to starvation conditions. *PDP2* expression was increased in starved PDK4^wt/del^ and PDK4^del/del^ cells as compared to unstarved controls but still remained significantly reduced as compared to levels in unstarved PDK4^wt/wt^ cells **(**Fig. 3B**)**. *PDH* transcription levels were significantly higher in both PDK4^wt/del^ and PDK4^del/del^ cells as compared to PDK4^wt/wt^ cells in normal culture conditions **(**Fig. 3C**)**. Only the PDK4^wt/del^ displayed a significant difference (decrease) between unstarved and starved conditions.

### Variable PDH protein expression and phosphorylation in PDK4 deficiency

**Figure 4:**
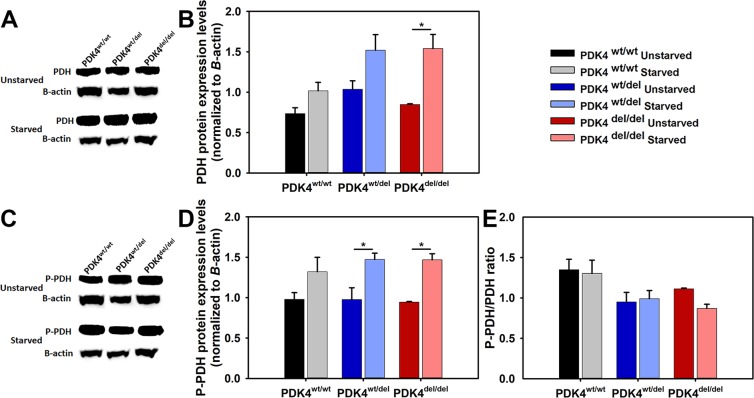
PDH levels and phosphorylation status in response to starvation. (**A**) WB image using an antibody that recognizes all PDH and (**B**) quantified PDH data normalized to B-actin, and (**C**) WB image using an antibody that recognizes PDH in its phosphorylated state P-PDH and (**D**) corresponding quantified data normalized to B-actin. Quantified data represent 3 WBs of cell lysates representing each of the three groups PDK4^wt/wt^, PDK4^wt/del^, and PDK4^del/del^ cells in unstarved and starved conditions. (**E**) Bar graph showing the ratio of P-PDH to PDH for each sample and condition. Full WB images are provided in supplemental data. (Data presented as mean + std. err. *p < 0.05).

Using a PDH antibody that recognizes the protein regardless of phosphorylation status western blots were performed to compare PDH protein levels between the different genotypes in unstarved and starved conditions **(**Fig. 4A**)**. The quantified results showed a significant increase in PDH protein expression in the 24 hour starved PDK4^del/del^ cells as compared to unstarved controls but no other differences were observed in cells from the other genotypes **(**Fig. 4B**)**. Next, an antibody that recognizes phosphorylated PDH was used to perform western blots to determine levels of the inactivated (phosphorylated) form of PDH (P-PDH) **(**Fig. 4C**)**. When quantified, the results showed that while all genotypes showed a trend towards more P-PDH under starvation conditions, this phenomenon was significant in only the PDK4^wt/del^ and PDK4^del/del^ cells **(**Fig. 4D**)**. Finally, we compared ratios of P-PDH to total PDH in cells from all genotypes under both normal and starved conditions and found that while not significant there is a trend that suggests PDK4^wt/wt^ cells may have higher levels of the inactivated (phosphorylated) form of PDH than the PDK4^wt/del^ and PDK4^del/del^ cells in both normal and starvation culture conditions **(**Fig. 4E**)**.

### PDH activity and expression levels are upregulated in PDK4 deficiency

**Figure 5:**
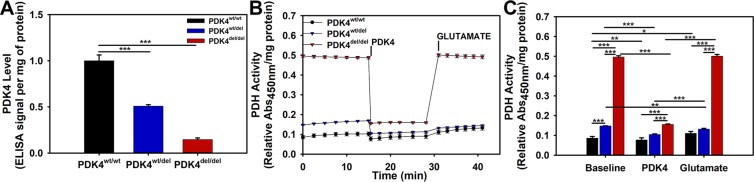
Relative PDK4 expression and PDH activities. (**A**) Relative PDK4 expression levels as determined by an ELISA, (**B**) PDH activity levels between cells representing the three different genotypes and their responses to PDK4 and subsequently, glutamate additions (x-axis indicates minutes time). (**C**) Quantified PDH activity data showing significant differences between cells and conditions. PDK4^wt/wt^, PDK4^wt/del^, and PDK4^del/del^ cells. (Data presented as mean + std. err. *p < 0.05, **p < 0.01, ***p < 0.001).

To evaluate the effects of PDK4 dysfunction on PDH activity levels, we first evaluated PDK4 expression levels by ELISA and confirmed a significant deficiency in both the PDK4^wt/del^ and PDK4^del/del^ cells **(**Fig. 5A**)**. Next, PDH activity was evaluated in cells representing each genotype using an ELISA assay. At baseline conditions, PDH activity was modest in PDK4^wt/wt^ cells, significantly elevated in PDK4^wt/del^ cells, and dramatically elevated in PDK4^del/del^ cells **(**Fig. 5B**)**. We performed a rescue experiment to confirm that this dramatically high level was a result of PDK4 deficiency. Purified PDK4 was added to all wells and resulted in a reduction of PDH activity that was significant for all cells regardless of mutation status **(**Fig. 5C**)**. The average percent decrease of PDH activity following PDK4 addition was 10.67% in PDK4^wt/wt^ cells, 31.52% in PDK4^wt/del^ cells, and 67.58% in PDK4^del/del^ cells. The subsequent addition of glutamate then restored PDH activity to baseline levels for all three groups **(**Fig. 5B,C**)**.

### PDK4 deficient cells display altered oxygen consumption rates

**Figure 6:**
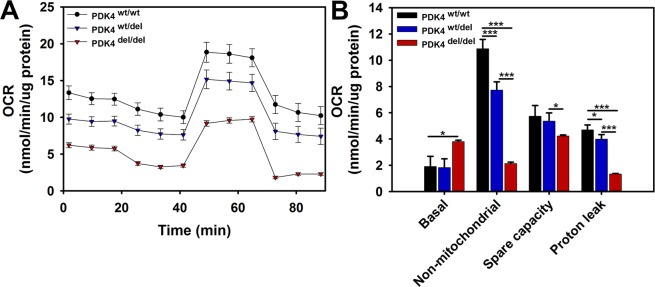
Extracellular Flux – Mitochondrial Stress Test. (**A**) Seahorse extracellular flux assay results profile showing relative responses to the addition of oligomycin, FCCP, and antimycin A/rotenone. (**B**) Quantified results representing relative OCR levels in PDK4^wt/wt^, PDK4^wt/del^, and PDK4^del/del^ cells (Data presented as mean + std. err. *p < 0.05, **p < 0.01, ***p < 0.001).

To compare oxygen consumption rates between the three groups of cells, the Seahorse Extracellular Flux Analyzer was used in conjunction with a Mitochondrial Stress Test assay. The results showed an overall reduced oxygen consumption rate profile for PDK4 deficient cells as compared to healthy controls **(**Fig. 6A**)**. While the basal oxygen consumption rate (OCR) was significantly increased in PDK4^del/del^ cells, non-mitochondrial OCR was significantly decreased **(**Fig. 6B**)**. Proton leak was also significantly increased in PDK4^del/del^ cells as compared to the other two groups **(**Fig. 6B**)**. Mitochondrial spare capacity, or the ability of cells to overcome increased stress is significantly decreased in the PDK4^del/del^ cells as compared to both other groups **(**Fig. 6B**)**. In sum, these data indicate that PDK4 deficiency significantly alters oxygen utilization by cells.

## Discussion

While PDK4’s crucial role in the maintenance of metabolic flexibility through phosphorylation and suppression of PDH activity is well described, the consequences of PDK4 deficiency, particularly in DPs, are less well understood. We sought to explore the mechanisms involved in DP PDK4 deficiency through characterization of primary dermal fibroblasts representing healthy, partial, and complete PDK4 deficiency. Specifically, we wanted to define differences in how the cells responded to starvation, measure relative compensatory behavior between genotypes based upon expression levels, and evaluate relevant enzymatic activities.

Our initial experiment focused on the impact of starvation on cell morphology and mitochondrial subcellular localization in DP fibroblasts representing each of the three genotypes. We observed overall altered cell morphology and increased perinuclear clustering of mitochondria in both PDK4^wt/del^ and PDK4^del/del^ cells as compared to healthy PDK4^wt/wt^ controls. These alterations suggest that PDK4 is required to maintain a regular cell shape and cytoskeletal structure. Interestingly, while cellular circularity was unchanged in PDK4^wt/wt^ cells following starvation, PDK4^wt/del^ cells significantly increase circularity, while PDK4^del/del^ cells become elongated and less circular. In all, these data demonstrate the impact of PDK4 deficiency on cell structure during starvation conditions.

Further support for the relationship between altered cellular structure and apoptosis is provided by our data showing increased perinuclear clustering of mitochondria in PDK4 deficient cells. In general, perinuclear localization of mitochondria is a sign of cell stress under various disease states or environmental conditions and this translocation has been shown to be physiologically important and may facilitate mitochondrial stress signaling mechanisms^25,29,30^. Indeed, these data correlate well with a previous study designed to assess PDK4 deficient DP fibroblasts where conditions causing perinuclear localization of mitochondria resulted in the activation of intrinsic apoptosis^25^. These results support the notion that PDK4^wt/del^ and PDK4^del/del^ cells react differently than PDK4^wt/wt^ cells to adverse environmental conditions.

As PDK4 both regulates the entry of substrates into the citrate cycle and is regulated by its products, and as all four PDKs regulate PDH, we reasoned that there may also be an indirect cross-regulation of the various PDK isoforms. We were therefore curious to see how the presence of a PDK4 splice site mutation impacted transcription levels of 3 other PDK isozymes (*PDK1*, *PDK2*, and *PDK3*) as up or down regulation of these may indicate attempted cellular adaptations to stress through compensatory pathways. We observed an interesting decrease in *PDK1* transcription under starvation conditions in only the PDK4^wt/del^ and PDK4^del/del^ cells which counterintuitively suggests that instead of upregulation of another PDK, there is perhaps an exaggerated impact on general PDK expression when one is deficient. This may be due to feed-back loop information as when PDK4 is functional, a highly active PDH suggests abundant glucose availability. In contrast, *PDK2* isozyme levels were significantly increased in all three cell types under starvation conditions which does support the idea that these cells are perhaps making some attempt to activate compensatory mechanisms. *PDK3* transcription was unique in that while both deficient lines displayed lower overall levels as compared to healthy controls, both PDK4^wt/wt^ and PDK4^del/del^ cells showed similar profiles with significant decreases under starvation conditions. Finally, PDK4^wt/del^ cells showed a significant increase in *PDK3* transcription which may represent a subtle but key compensatory pathway available to that genotype which enhances its ability to adapt to starvation as compared to PDK4^del/del^ cells.

We also assessed *PDK4* transcription across the three genotypes using a primer/probe set lying upstream of the splice site mutation that would identify transcripts regardless of mutation status. Unsurprisingly, there was a decrease in both affected genotypes as compared to healthy controls. However, the decrease in PDK4 transcripts in heterozygous cells even under starvation conditions was more dramatic than expected and again, likely suggests the deficient cells react to feed-back loop information that enhances the problem.

Our evaluations of PDP1 and PDP2 transcription levels revealed a significant increase in both the PDK4^wt/del^ and PDK4^del/del^ cells under starvation conditions and in fact, the PDK4^del/del^ cells display not only a switch in profile direction upon starvation but also a significant increase in *PDP1* transcription as compared to both PDK4^wt/wt^ cells. Such conditions further exacerbate the underlying PDK4 deficiency by creating a situation in which the PDH is even more likely to become dephosphorylated and thus activated, than usual.

To complement these data, we also assessed *PDH* transcription levels in cells representing the three genotypes and found that while each line behaved differently, in typical culture conditions there is a significant increase in *PDH* transcripts in both deficient lines as compared to healthy controls. This too, enhances the opportunity for either various PDKs or PDPs to influence PDH function within the milieu of PDK4 deficiency. Further evaluation of PDH protein expression and phosphorylation status were inconclusive but ratiometric comparisons of phosphorylated PDH (inactive) to dephosphorylated PDH (activated) suggest a trend where the is less activated PDH in healthy controls as compared to either of the PDK4 deficient lines. The specific antibody used Phospho-PDHA1/PDHA2 detects PDHA1/PDHA2 only when phosphorylated at Ser293 and/or Ser291. These data also indicate that something (likely another PDK isozyme) is managing to phosphorylate PDH in PDK4 deficient cells to some extent or perhaps the PDH remains phosphorylated longer due to decreased PDP2 levels.

It is important to note that different PDK isoforms have specific affinities for different PDH phosphorylation sites. The three phosphorylation sites are located in the PDH E1-alpha subunit, and the four PDK isozymes allosterically inhibit protein structure in different ways^31^. Site 1 phosphorylation interferes with E1’s interaction with dihydrolipoamide acetyltransferase, while site 3 phosphorylation likely impedes interaction of E1 with the coenzyme thiamine pyrophosphate (TPP). While other *in vitro* studies have shown that all PDK isoforms phosphorylate sites 1 and 2, site 3 is only phosphorylated by PDK1^31^. Thus, for proper evaluation of the effects of PDK4 deficiency on the PDH complex, it was important that the antibody we used recognized phosphorylation sites 1 and 2.

While PDK4 protein levels were found to be significantly reduced in PDK4 deficient lines, the relative impact of all these factors on actual PDH activity in cells representing the three different genotypes helped to elucidate the full impact of this disorder. PDH activity is significantly increased in PDK4^del/del^ cells at baseline, following addition of PDK4, and subsequently, in the presence of additional glutamate. While the PDK4^wt/del^ cells display some increase in PDH activity as compared to healthy controls, it is a far more subtle response.

Finally, as oxygen consumption measurements are useful indicators to assess overall mitochondrial function, we performed mitochondrial stress tests using the Seahorse Extracellular Flux system to evaluate cells representing all three genotypes. While our results show an overall lower O_2_ consumption profile for both PDK4^wt/del^ and PDK4^del/del^ cells as compared to healthy controls, the data also show dramatically decreased non-mitochondrial O_2_ consumption in PDK4^del/del^ cells. Taken together, these two outcomes suggest a general decrease in cellular metabolism in PDK4 deficiency. Spare capacity is a good general indicator of overall mitochondrial health and was found to be decreased in PDK4^del/del^ cells as compared to PDK4^wt/del^ cells revealing yet another difference in how these two genotypes respond to stress. Additionally, proton leak was found to be significantly reduced in PDK4^del/del^ cells as compared to the other two genotypes. While there is some debate as to whether proton leak is a positive or negative, the current prevailing theory is that when present at appropriate levels, proton leak decreases mitochondrial reactive oxygen species (ROS) production^32^. As ROS is a necessary by-product of healthy ETC function, there may be a greater need for this protective role in cells with more robust ETC activities such as those observed in the PDK4^wt/wt^ and PDK4^wt/del^ cells evaluated in this study.

In sum, this study links PDK4 deficiency to the constitutive activation of PDH and strongly suggests that at the cellular level, PDK4 deficient DPs face impaired metabolic flexibility. Strategies to mitigate this deficiency in DPs are currently in early research stages and include modified diets and nutritional supplements designed precisely for these dogs. For example, as PDK4 functions at the interface of glucose and lipid metabolism, there may be potential benefits from a ketogenic diet for PDK4 deficient DPs. Alternatively, we may find that a high carbohydrate diet would sufficiently provide the fuel necessary to support a constantly active PDH and avoid fuel source depletion that may contribute in part to the eventual development of DCM in these DPs.

Importantly, these evaluations need to be tested in translational models to confirm efficacy and support clinical translation prior to placing these canine patients at potential risk for unintended adverse events. Our study using fibroblasts supports the inclusion of PDK4 deficiency as an early biomarker for the detection of DP populations that are at risk for the development of DCM. While dermal fibroblasts and the cardiomyocytes that are implicated in DCM are very different cell types, a previous report demonstrated altered mitochondrial structure in heart muscle from PDK4 deficient dogs, we found that all four PDK isoforms found in the heart are also expressed in our dermal fibroblasts, and starvation has previously been shown to specifically upregulate PDK2 and PDK4 in the heart and we observed this same response in our fibroblasts^8,33–35^. Thus, while fibroblasts may not accurately model mechanisms such as contractility, ion handling, and metabolic flexibility, that are crucial to healthy cardiomyocyte function, we have demonstrated that these cells can be useful for exploring the impact of PDK4 deficiency on pyruvate dehydrogenase activity.

Although assessments of the impact of different fuel source supplements (fatty acids, carbohydrates, etc.) would ideally be evaluated in cardiomyocytes isolated directly from DPs, these cells are extremely difficult to procure due to the often sudden nature of death in this breed. Our future studies include plans to reprogram cells from DPs into induced pluripotent stem cells (iPSCs) which can then be differentiated into cardiomyocytes. Such a model would provide a useful platform for more thorough investigations into disease mechanisms of PDK4 deficiency as well as the testing of nutritional supplements, pharmaceuticals, and potentially CRISPR-Cas based genome editing approaches to mitigate the onset and progression of DCM in DPs.

## Materials and Methods

### Approval and accordance

This study was conducted in accordance with the guidelines of the Animal Care and Use Committee (IACUC) at the University of Florida. The study was approved by the IACUC committee #201405165. Written informed consent authorizing study participation was obtained from each owner of the DPs used in this study. Doberman Pinchers deemed healthy on physical examination were enrolled in this study; there were a total of 18 canines, including 6 PDK4^wt/wt^, 6 PDK4^wt/del^ and 6 PDK4^del/del^ animals used for the study and averaged where appropriate. They were all client-owned dogs returned to their family shortly after the biopsy procedure was performed.

### Skin biopsy procedure

The inguinal area was clipped and a local anesthetic (0.3 mL lidocaine) was injected, followed by three alternating chlorhexidine and alcohol scrubs disinfection. A skin biopsy at approximately five centimeters caudal to the femoral artery was performed. The skin surrounding the biopsy site was stretched and a 3-4 mm punch biopsy instrument (Paramount Biopsy Punch) was held vertically over the skin and rotated downward using a twirling motion. The cylindrical skin specimen was elevated and the specimen cut with scissors from the subcutaneous tissues. The small biopsy site was closed with surgical tissue glue (Glustitch PeriAcryl® Tissue Adhesive). A telfa pad was applied over the skin biopsy site after the procedure. No complications were noted on any of the subjects.

### Fibroblast cell culture

Primary dermal fibroblasts were harvested from skin biopsies of DPs and cultured in DMEM with 10% fetal bovine serum (FBS), 1% Penicillin-Streptomycin and Amphotericin-B at 37 °C and 5% CO_2_. For starvation experiments, fibroblasts were cultured in medium depleted of glucose and FBS for 24 and 48 hours. Viability assays were performed using Trypan blue assays.

### Taqman gene expression analysis

Total RNA was extracted from fibroblasts using Quick-RNA Mini-prep kit (Zymo Research, Irvine, CA, USA) according to the manufacturer’s protocol. Total RNA was converted to cDNA using the High Capacity RNA-to-cDNA Kit (Applied Biosystems, Carlsbad, CA, USA). Real Time qPCR was performed using custom made primers (Table 1) and primers designed based upon previous studies (Table 2) on a StepOne™/StepOnePlus™ Real-Time PCR System (Applied Biosystems). The relative fold change expression levels were calculated by the ΔΔCT method.

**Table 1:** Custom made primers used for quantitative real-time PCR.

Primer	Sequence (5′-3′)	Location
HPRT1 Forward	AGC/TTGCTGGTGAAAAGGAC	exon 5/6
HPRT1 Reverse	TTATAGTCAAGGGCATATCC	exon 7
PDK4 Exon 8 Forward	AATGCAATGAGGGCAACAGTTGAA	5′-end of exon 8
PDK4 Exon 9 Reverse	GTTTCCTCGTAAGGCCCTTAATAG	3′-end of exon 9
PDK4 Exon 10 Forward	GCTGGTTTTGGTTATGGCTTACCA	5′-end of exon 10
PDK4 Exon 11 Reverse	AAAGGACAACATTATTTTATAA	3′-end of exon 11

Sequences are shown in 5′-3′ direction. Primers for the housekeeping gene HPRT are taken from Brinkhof *et al*.^36^ PDK4 primers were always taken from the 5′-end of the first exon to the 3′-end of the second exon. A forward slash marks a boundary between two exons.

**Table 2:** Designed primers based on previous studies used for quantitative real-time PCR.

Primer	Company
PDK1	Thermo Scientific #Cf02700627_g1
PDK2	Thermo Scientific #Cf02700630_g1
PDK3	Thermo Scientific #Cf01017871_m1
PDP1	Thermo Scientific #Cf02711767_s1
PDP2	Thermo Scientific #Cf02712806_s1
PDH	Thermo Scientific #Cf02701742_m1

### Immunofluorescence and microscopy

Representative fibroblasts from each genotype (PDK4^wt/wt^, PDK4^wt/del^, and PDK4^del/del^) were seeded onto coverslips, grown for 24 hr, rinsed three times with 1x PBS, and fixed with 4% paraformaldehyde. All conditions were stained with antibodies (Table 3) in the following dilutions: 1) anti-TOMM20 (1:200), 2) anti-Phalloidin (1:50) and mounted on microscope slides for imaging with medium containing DAPI.

**Table 3:** Antibodies used in this study for immunohistochemistry.

Antibody	Company
Anti-TOMM20	Sigma Aldrich #HPA011562
Anti-Phalloidin	Life Tech #A12379

### Protein quantification and Western blot analyses

Fibroblasts were lysed in RIPA lysis buffer containing protease and phosphatase inhibitors. Protein concentrations were determined using the DC Protein Assay kit (BioRad). Samples were resolved on 5–10% bis-tris polyacrylamide denaturing gels and transferred to nitrocellulose membranes. Membranes were incubated overnight at 4 °C with primary antibodies (Table 4) in the following dilutions: (1) anti-PDH (1:1000), (2) anti-P-PDH (1:1000), and (3) anti-β-actin (1:5000). Membranes were washed with 1xTris-buffered saline with Tween (TBS-T), incubated with secondary antibodies for 1 hour, and washed with TBS-T. The bands detection were performed using Amershan ECL Prime Western Blotting Detection Reagent (GE Healthcare Life Science). Representative images from cropped blots are presented in **(**Fig. 4**)**. Full blots used to generate bar graph data are provided in **(**Supplemental Fig. 1**)**.

**Table 4:** Antibodies used in this study for Western blot.

Antibody	Company
Anti-PDH	Invitrogen #459400
Anti-P-PDH	Invitrogen #PA5-64845
Anti-β-actin	Santa Cruz #sc47778

### PDH activity

PDH activity was measured with an ELISA kit following manufacturer’s instructions (Abcam, #ab110671). Protein extracts were incubated for 3 hours at room temperature on plates pre-coated with the PDH antibody. Following incubation, plates were emptied, washed twice with Stabilizer Buffer and incubated for 1 hour at room temperature with Detector Antibody. PDH activity was determined by colorimetric measurement every 36 seconds at 450 nm, and normalized to the total protein. The measurements were acquired following steps: (1) Without substrate addition (Baseline), (2) With subsequent addition of human recombinant PDK4 (OriGene-TP301656), and (3) With subsequent addition of glutamate.

### PDK4 level

Relative PDK4 expression levels were measured with a PDK4 ELISA kit per manufacturer’s instructions (Abcam, #ab126582).

### Extracellular flux assay - mitochondrial stress test

Cells were seeded at a density of 50,000 cells per well in XF96-well microplate (Seahorse Bioscience). Cells were incubated for 24 hr into standard growth medium in a humidified incubator at 37 °C with 5% CO_2_. After 24 h, the standard medium was exchanged by Krebs-Henseleit buffer with 0.5% Bovine Serum Albumine (BSA), 50 mM carnitine, 200 µm palmitate, 1 mM pyruvate, 2 mM glutamine, and 10 mM glucose. Subsequently, fibroblasts were incubated for 1 hour at 37 °C without CO_2_. OCR and ECAR were determined using XF Cell Mito Stress Assay (Seahorse Bioscience) following additions of: oligomycin (1 µM), carbonylcyanide-p-trifluoromethoxyphenylhydrazone (FCCP) (1.5 µM) and rotenone/antimycin A (1 µM). Measurements were repeated in triplicate. Data were analyzed using Wave Desktop Software (Seahorse Bioscience) following the manufacturer’s instructions and normalized to protein levels.

### Morphology analysis

Analysis of cell shape and mitochondrial distribution was performed using ImageJ (NIH, Bethesda). To measure cellular circularity we used the ratio between a cell’s area and the square of its perimeter, multiplied by 4π: Circularity = 4π · area/(perimeter)^2^. For a circle, the circularity is ‘1’, while it is ‘0’ for a line. To analyze mitochondrial distribution (ratios of perinuclear to peripheral), a circle was drawn at a distance of 2 µm from every nucleus. For each cell, the amount of TOMM20 signal was assessed within (perinuclear) and outside of (peripheral) the circle surrounding the nucleus. For each measurement, ≥20 cells were assessed for each condition and genotype.

### Statistical analysis

Data analyses and graph creations were performed using Sigma Plot Software. There were 3–6 biological replicates for each condition and experiment and plate-based and blot assays were run in triplicate. Descriptive and inferential statistics, such as paired t-tests, were performed where appropriate.

## Supplementary information

Dataset 1.

## Data Availability

The data and reagents will be available upon request to senior author C.A.P.
