# Supplementary material for: Functional Consequences of PDK4 Deficiency in Doberman Pinscher Fibroblasts
Source: Sci Rep. 2020 Mar 3;10:3930. doi: 10.1038/s41598-020-60879-6 (PMC7054397; doi:10.1038/s41598-020-60879-6)

Functional Consequences of PDK4 Deficiency in Doberman Pinscher Fibroblasts

Luiz Bolfer[1], Amara H. Estrada[1], Chelsea Larkin[1,3], Thomas J. Conlon[2], Francisco Lourenco[1], Kathryn Taggart[1], Silveli Suzuki-Hatano[3], Christina A. Pacak[1,3,4*]

Supplemental Figure 1: The whole gel images used to generate data for figure 4 in the main body of the manuscript.

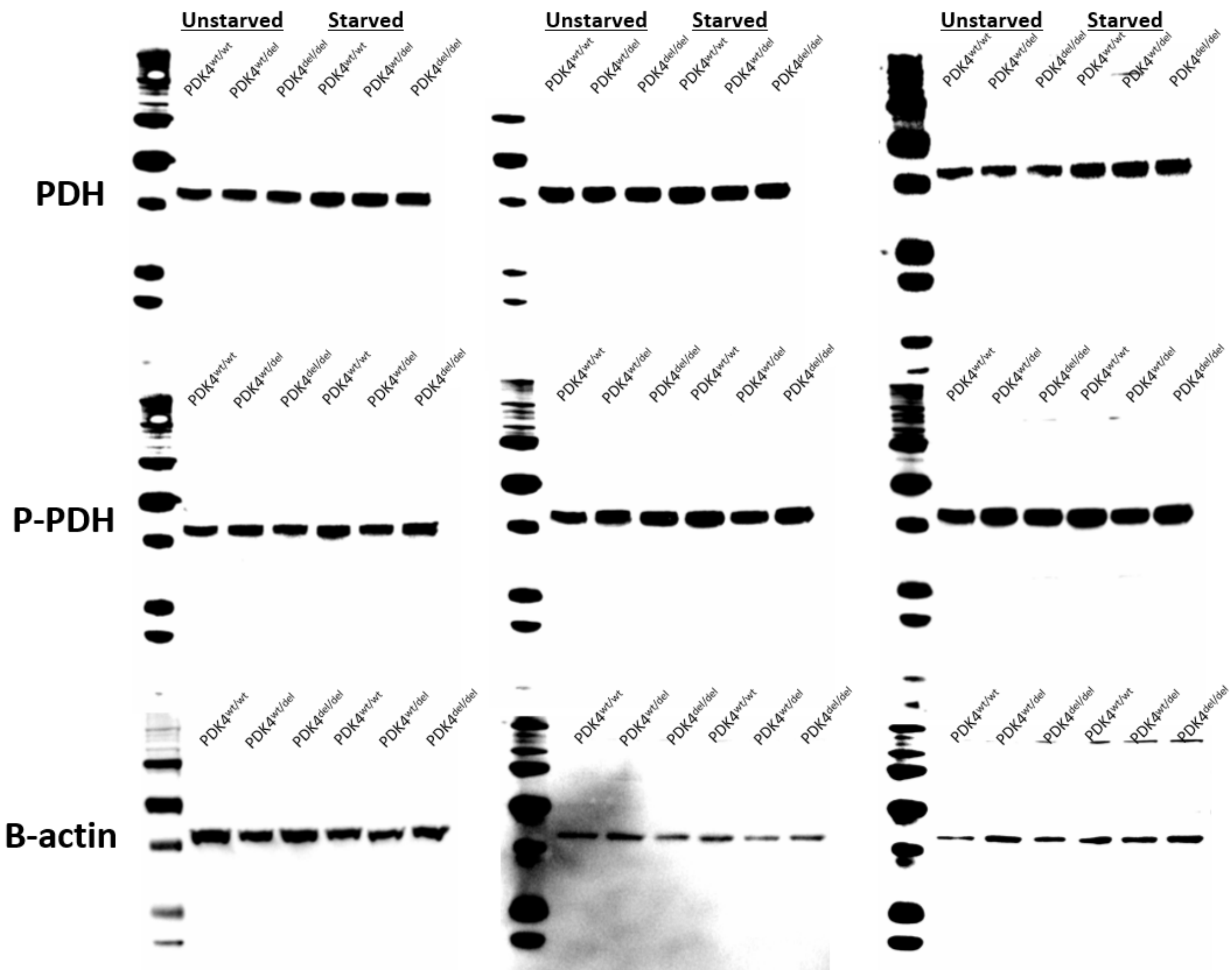

Supplement: Supplementary file 1 — Dataset 1. [file 41598_2020_60879_MOESM1_ESM.pdf]
